# Supplementary material for: Geologically younger ecosystems are more dependent on soil biodiversity for supporting function
Source: Nat Commun. 2024 May 16;15:4141. doi: 10.1038/s41467-024-48289-y (PMC11099028; doi:10.1038/s41467-024-48289-y)
Supplement: Supplementary file 1 — Supplementary Information [file 41467_2024_48289_MOESM1_ESM.pdf]

Supplementary Materials

Geologically younger ecosystems are more dependent on soil biodiversity for supporting function

Jiao Feng, Yu-Rong Liu, David Eldridge, Qiaoyun Huang, Wenfeng Tan, Manuel Delgado-Baquerizo

Corresponding authors: Yu-Rong Liu; Email: yrliu@mail.hzau.edu.cn;
Manuel Delgado-Baquerizo; Email: m.delgado.baquerizo@csic.es

This file includes.
Figures S1 to S15
Table S1 to S2

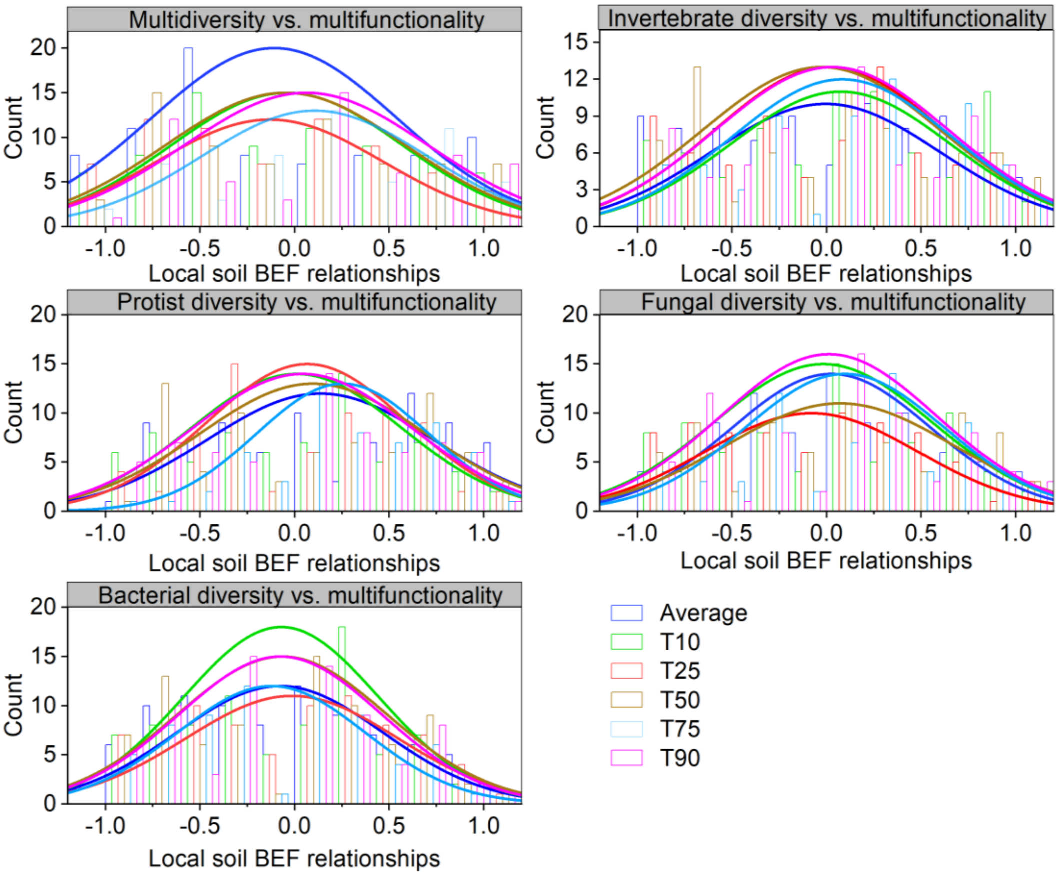

Figure S1 | Distributions of local soil biodiversity and ecosystem function (BEF) relationships from 87 globally distributes sites across 16 soil chronosequences. Average lines represent averaging Multifunctionality calculated from averaging all individual 13 individual functions after 0-1 standardization (Methods); T10, T25, T50, T75 and T90, represents multifunctionality calculated at different threshold of 10%, 25%, 75% and 90%, respectively. The results for Skewness, Kurtosis, and normality tests are shown in Table S2. Source data are provided as a Source Data file.

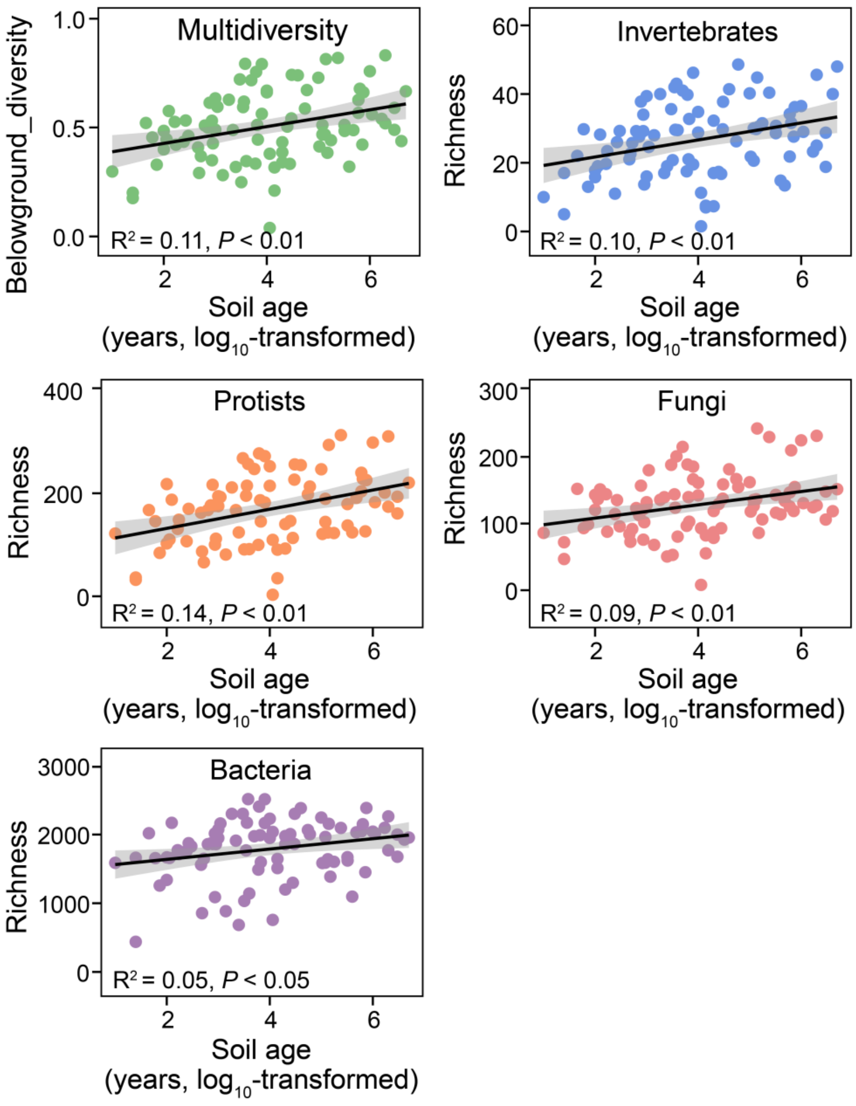

Figure S2 | The relationships between multitrophic soil biodiversity (including multidiversity, invertebrates, protists, fungi, and bacteria) and soil age. Multidiversity represents the averaging standardized scores (0-1 normalization) of diversity of all soil groups. The error bands surrounding the regression lines represent the 95% confidence interval of the correlation. Exact *P*-value and Source data are provided as a Source Data file.

(A)

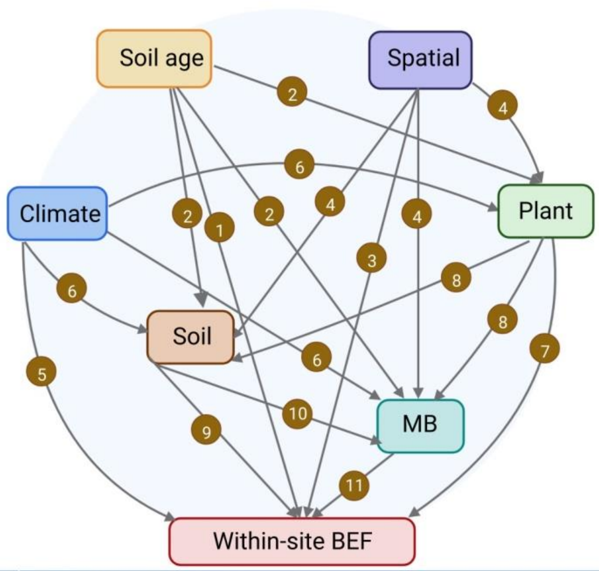

(B)

	Associations	Rationale
❶	Age→Within-site BEF	Soil age or pedogenesis is expected to affect soil BEF between multidiversity and multifunctionality as ecosystem proceeds.
❷	Age→Others	Soil age regulates microbial biomass, plant and edaphic attributes. For instance, soil age commonly lead to predictable changes in soil geochemical properties, such as SOC, pH, soil texture etc.
❸	Spatial→Within-site BEF	Spatial location could be crucial to soil BEF relationships due to highly heterogeneities of both biodiversity and ecosystem functions.
❹	Spatial→Others	Spatial locations are known to affect microbial biomass, soil properties, and distributions of plant communities.
❺	Climate→Within -site BEF	Climate factors such as mean annual precipitation and temperature is known to affect biodiversity of different soil microbial groups and also contribute to changes in their metabolic activities and associated ecosystem functions.
❻	Climate→Others	Climate factors are known to be important drivers for soil properties and abundance and diversity of both above- and below-ground communities.
❼	Plant→Within-site BEF	Plant communities could influence soil BEF relationships by their effects on quality and quantity of litters inputted.
❽	Plant→Others	Plant is known to affect soil properties and belowground communities such as through litter/root residues inputs or affect the physical structure of soil.
❾	Soil→Within-site BEF	Soil properties such as SOC, pH could affect soil BEF relationships by influencing the compensatory effects and ecosystem redundancy of different soil species.
❿	Soil→MB	Soil properties such as SOC and pH are known to influence the production of microbial biomass
⓫	MB→Within-site BEF	The microbial biomass are typical factors driving ecological processes, and thus may affect the necessity of soil biodiversity to sustain functions

Figure S3 | Conceptual model evaluating potential drivers for the local (within-site) soil multidiversity and ecosystem function (BEF) relationships. (A) A priori structural equation modeling (SEM) aimed to evaluate the direct and indirect associations of soil age and the local BEF relationship between multidiveristy and multifunctionality after considering other key ecological factors (including spatial, climatic, edaphic attributes and microbial network parameters). (B) Explanations for each association in the priori model. Multidiversity represents the diversity of four groups of soil organisms (invertebrates, protists, fungi and bacteria); Multifunctionality is the averaging of all 13 individual functions after 0-1 standardization (Methods). MB, microbial biomass.

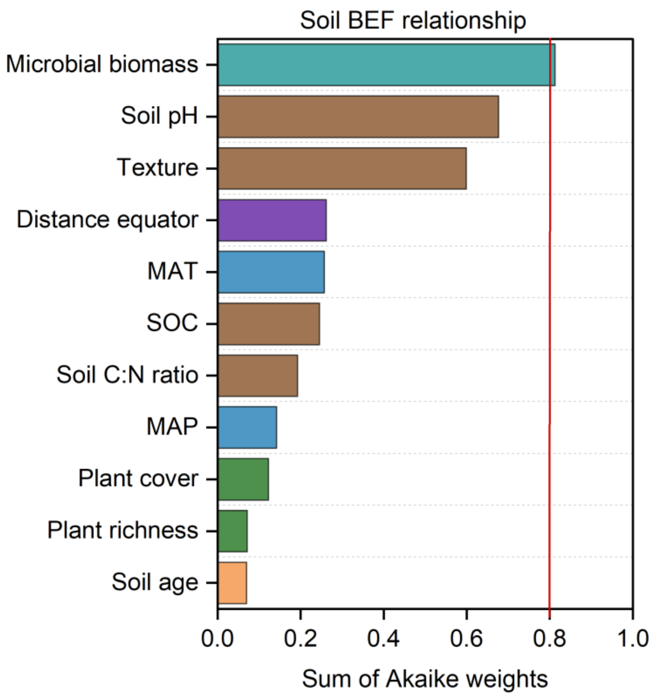

Figure S4 | Relative importance of different variables regulating the relationship between within-site multidiversity and ecosystem function (BEF). Multidiversity represents averaging biodiversity of four groups of soil organisms, including invertebrates, protists, fungi and bacteria. Multifunctionality, the averaging value of 13 individual functions after 0-1 standardization. Relative importance is shown according to the sum of Akaike weights of model selection. A cutoff of 0.80 was set to differentiate the important versus non-essential predictors. MAT, mean annual temperature; MAP, mean annual precipitation; SOC, soil organic C. Source data are provided as a Source Data file.

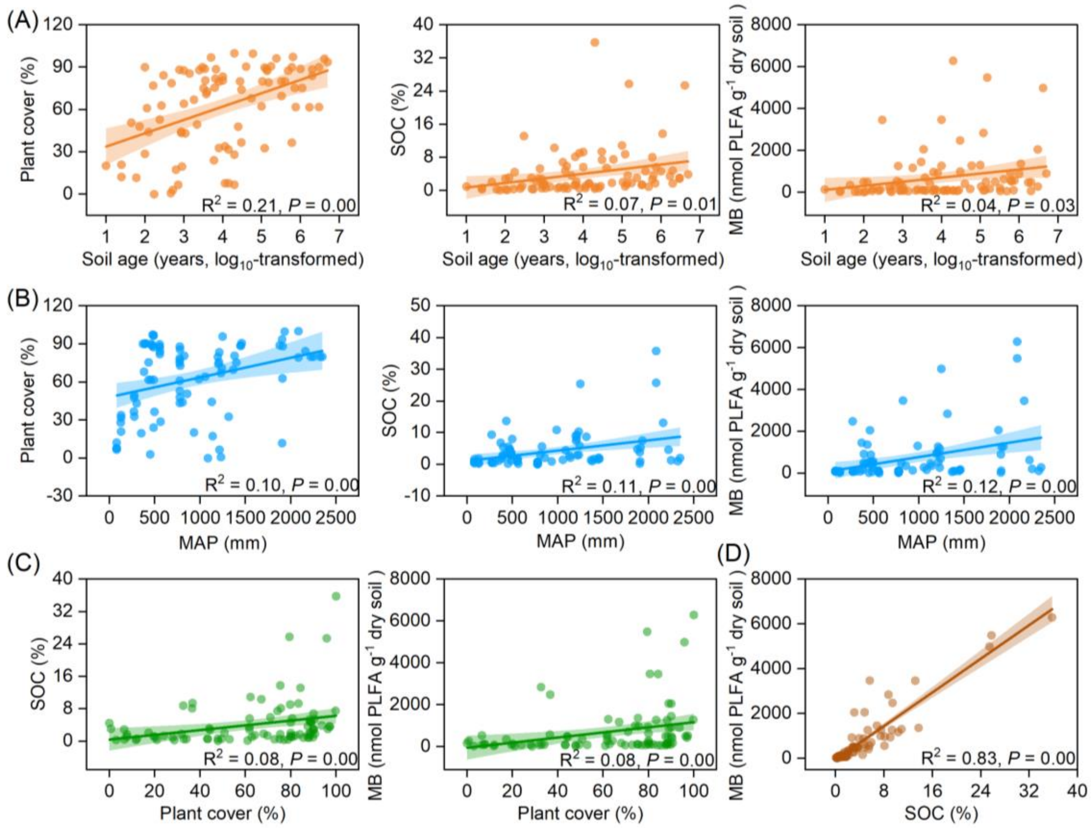

Figure S5 | Variations in plant cover, soil organic carbon (SOC) content and microbial biomass (MB) as soil age and rainfall changes. (A) Relationships of plant cover, SOC and MB as soil ages. (B) Variations of plant cover, SOC and MB with mean annual precipitation (MAP). (C) Relationships of plant cover with SOC and MB. (D) Relationship between SOC and MB. The error bands surrounding the regression lines represent the 95% confidence interval of the correlation. Source data are provided as a Source Data file.

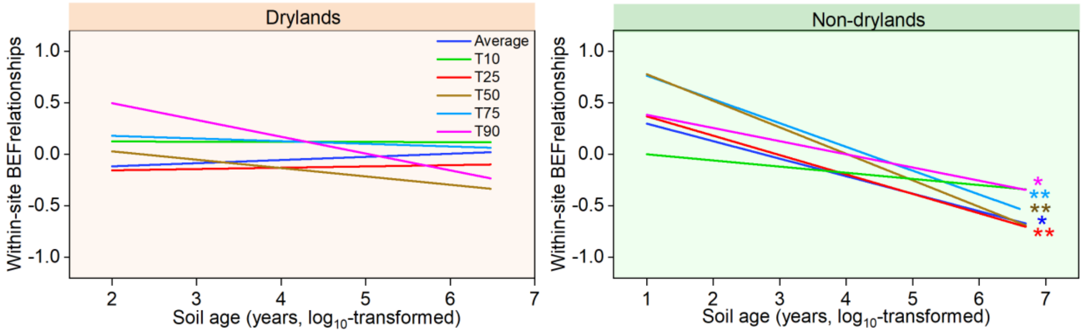

Figure S6 | Within-site soil relationship between soil multidiversity and function (BEF) along chronosequences in drylands and non-drylands ecosystems. Exact *P*-value and Source data are provided as a Source Data file. A two-sided test was used to assess the significance of the correlation analysis, with a threshold of *P*-value < 0.05 (*) and < 0.01 (**), respectively. Exact *P*-value and source data are provided as a Source Data file.

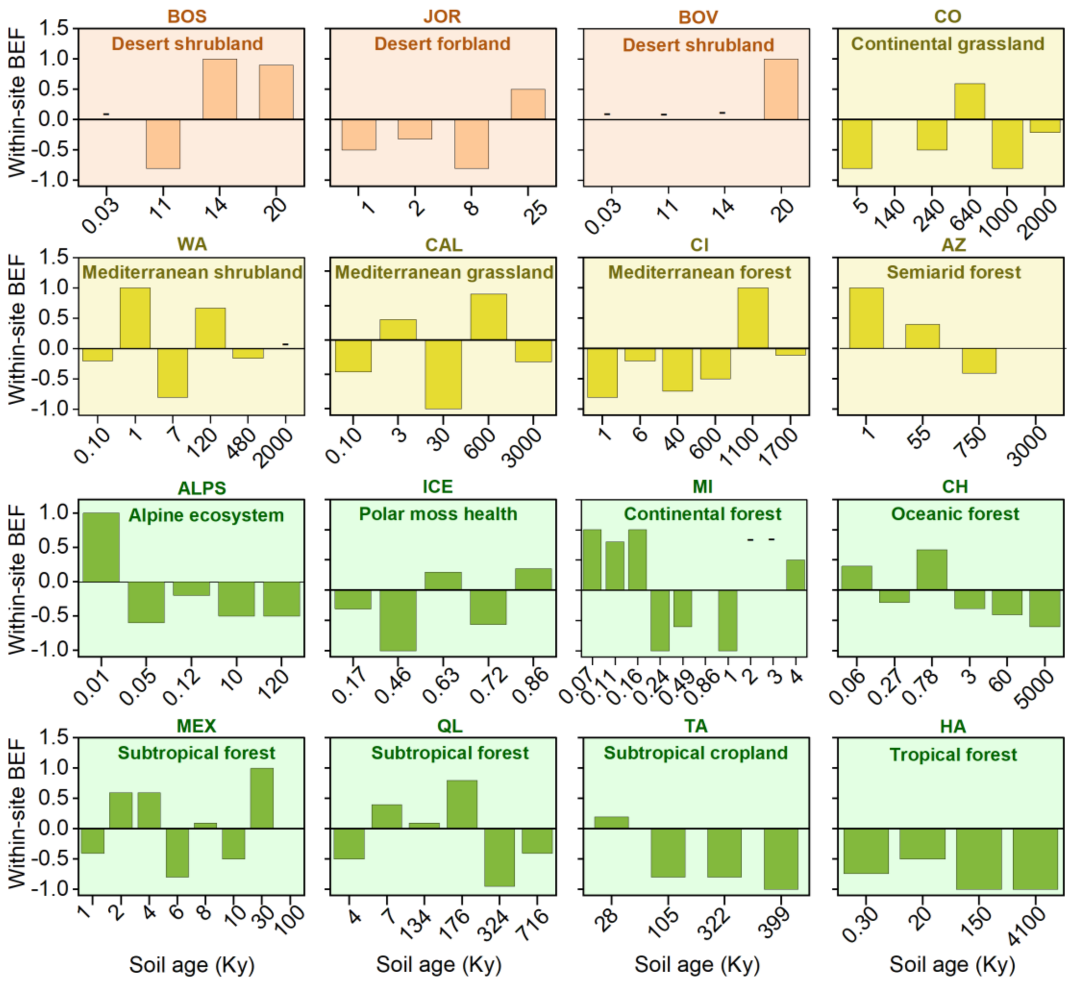

Figure S7 | Changes in local (within-site) soil biodiversity and ecosystem function (BEF) relationships during ecosystem development across 16 globally distributed soil chronosequences. The color of column indicates different climatic zone: orange, extremely arid regions; yellow, semi-arid regions; green, non-dryland ecosystems. BOS, Cojiri, Bolivia; JOR, Jornada Desert, USA; BOV, Chiar Kkollu, Bolivia; CO, Coal Creek, USA; WA, Jurien Bay, Western Austrilia; CAL, Merced, USA; CI, La Palma, Spain; AZ, SAGA, USA; ALPS, Alps, Austria; ICE, Mt. Hekla, Iceland; MI, Lake Michigan, USA; CH, Conguillio, Chile; MEX, Chichinautzin; QL, Queensland, Australia; TA, Taiwan; HA, Hawaii, USA. A "-" symbol indicates no valid BEF correlation coefficients because the associated soil samples did not pass the cut-off after rarefaction for belowground biodiversity; Ky, 1000 years. Source data are provided as a Source Data file.

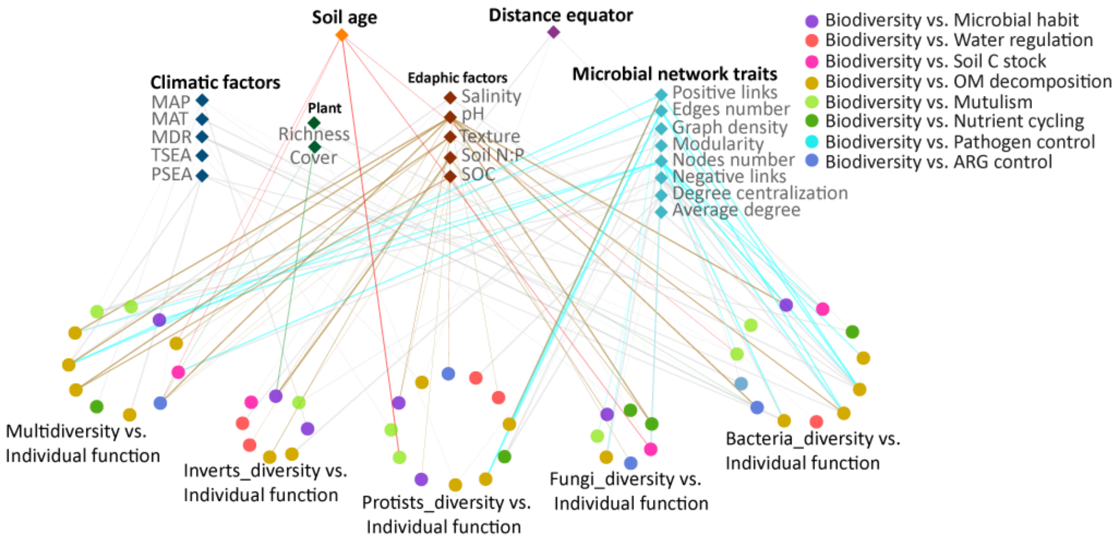

Figure S8 | Factors relating to the relationships between soil biodiversity and individual ecosystem functions. Multidiversity represents averaging biodiversity of four groups of soil organisms, including invertebrates, protists, fungi and bacteria. Circles with different colors represent the relationships between soil biodiversity and different categories of ecosystem functions. Source data are provided as a Source Data file.

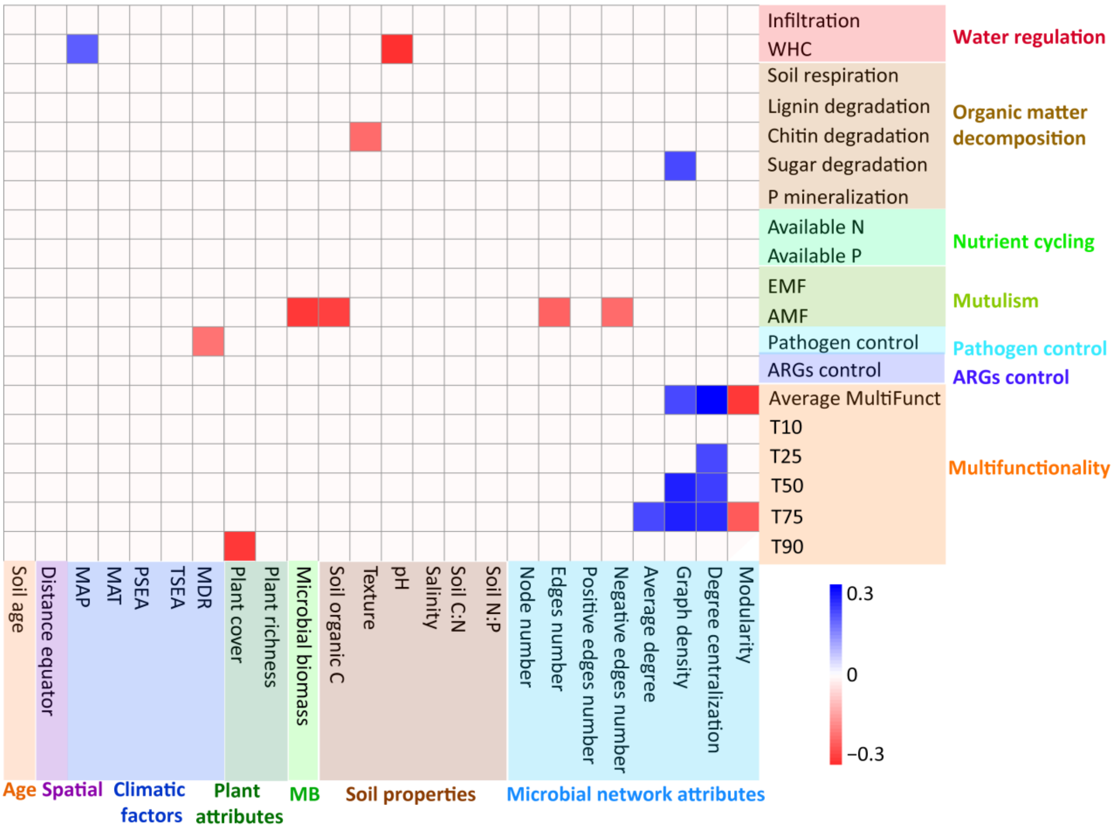

Figure S9 | Potential environmental factors regulating the local (within-site) relationship between invertebrates biodiversity and multifunctionality. MAP, mean annual precipitation; MAT, mean annual temperature; PSEA, precipitation seasonality; TSEA, temperature seasonality; MDR, mean diurnal range. WHC, water holding capacity; EMF, Ectomycorrhizal fungi; AMF, Arbuscular mycorrhizal fungi; ARGs, antibiotic resistance genes; MB, microbial biomass. Average MultiFunct represents Multifunctionality qualified by averaging all 13 individual functions after 0-1 standardization (Methods); T10, T25, T50, T75 and T90, represents multifunctionality quantified at threshold of 10%, 25%, 75% and 90%, respectively. Source data are provided as a Source Data file.

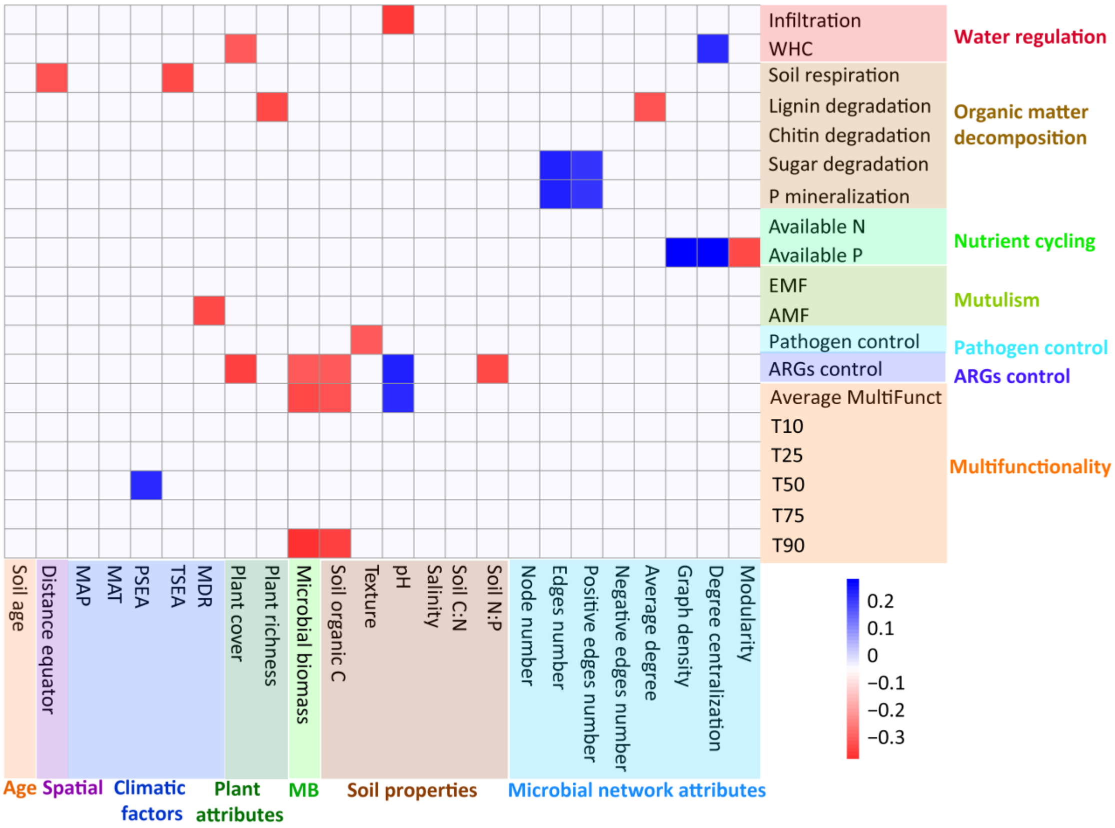

Figure S10 | Potential environmental factors regulating the local (within-site) relationship between biodiversity of protists and multifunctionality. MAP, mean annual precipitation; MAT, mean annual temperature; PSEA, precipitation seasonality; TSEA, temperature seasonality; MDR, mean diurnal range. WHC, water holding capacity; EMF, Ectomycorrhizal fungi; AMF, Arbuscular mycorrhizal fungi; MB, microbial biomass. ARGs, antibiotic resistance genes; Average MultiFunct represents Multifunctionality qualified by averaging all 13 individual functions after 0-1 standardization (Methods); T10, T25, T50, T75 and T90, represents multifunctionality quantified at threshold of 10%, 25%, 75% and 90%, respectively. Source data are provided as a Source Data file.

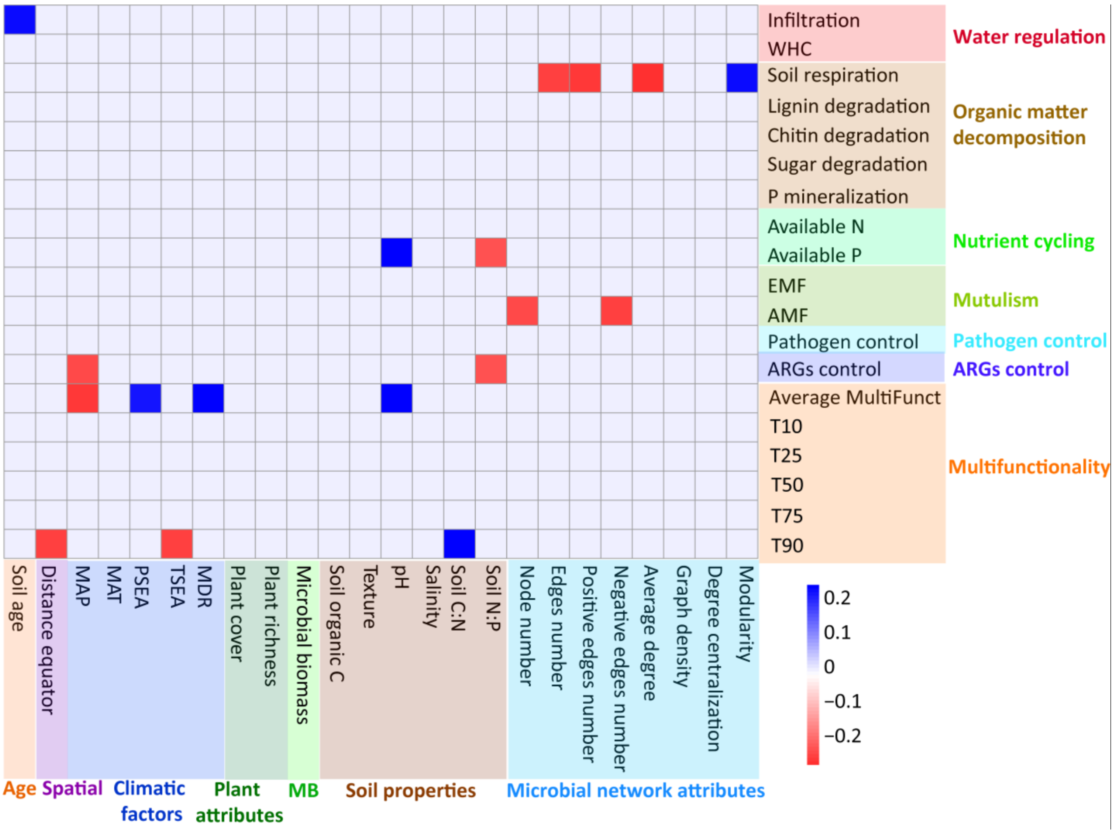

Figure S11 | Potential environmental factors regulating the local (within-site) relationship between biodiversity of fungi and multifunctionality. MAP, mean annual precipitation; MAT, mean annual temperature; PSEA, precipitation seasonality; TSEA, temperature seasonality; MDR, mean diurnal range. WHC, water holding capacity; EMF, Ectomycorrhizal fungi; AMF, Arbuscular mycorrhizal fungi; MB, microbial biomass. ARGs, antibiotic resistance genes; Average MultiFunct represents Multifunctionality qualified by averaging all 13 individual functions after 0-1 standardization (Methods); T10, T25, T50, T75 and T90, represents multifunctionality quantified at threshold of 10%, 25%, 75% and 90%, respectively. Source data are provided as a Source Data file.

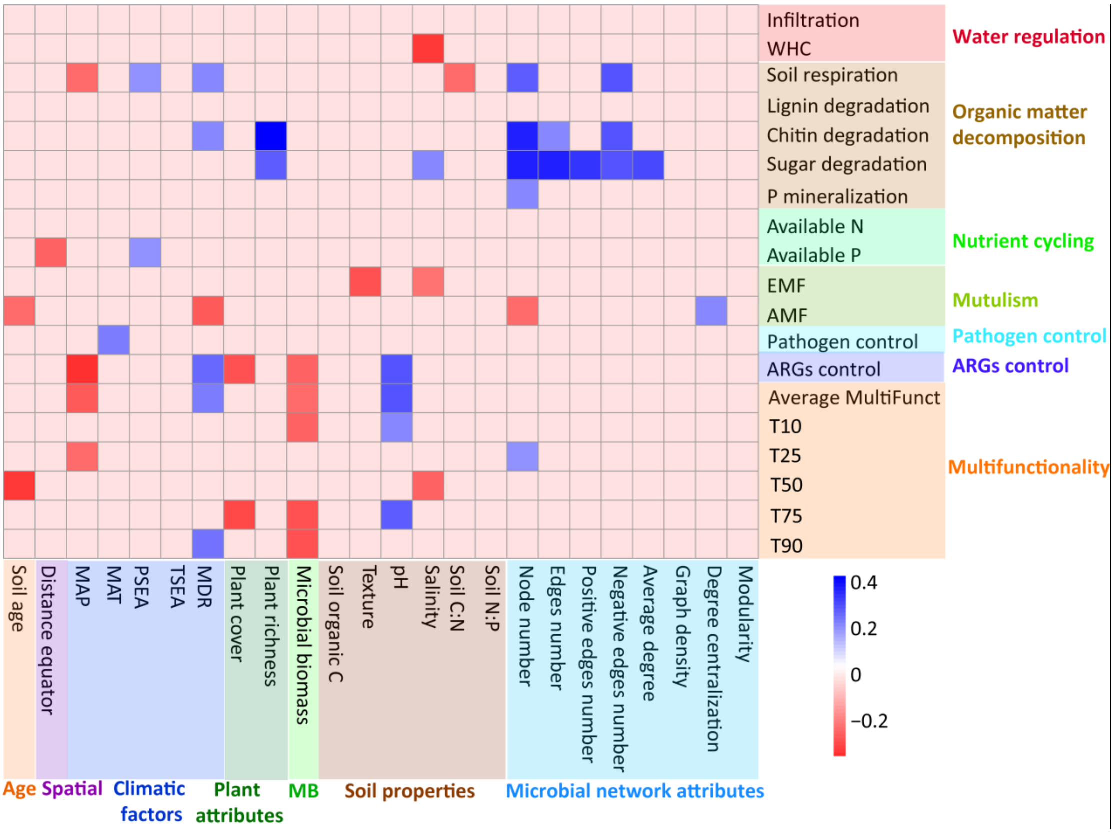

Figure S12 | Potential environmental factors regulating the local (within-site) relationship between biodiversity of bacteria and multifunctionality. MAP, mean annual precipitation; MAT, mean annual temperature; PSEA, precipitation seasonality; TSEA, temperature seasonality; MDR, mean diurnal range. WHC, water holding capacity; EMF, Ectomycorrhizal fungi; AMF, Arbuscular mycorrhizal fungi; MB, microbial biomass. ARGs, antibiotic resistance genes; Average MultiFunct represents Multifunctionality qualified by averaging all 13 individual functions after 0-1 standardization (Methods); T10, T25, T50, T75 and T90, represents multifunctionality quantified at threshold of 10%, 25%, 75% and 90%, respectively. Source data are provided as a Source Data file.

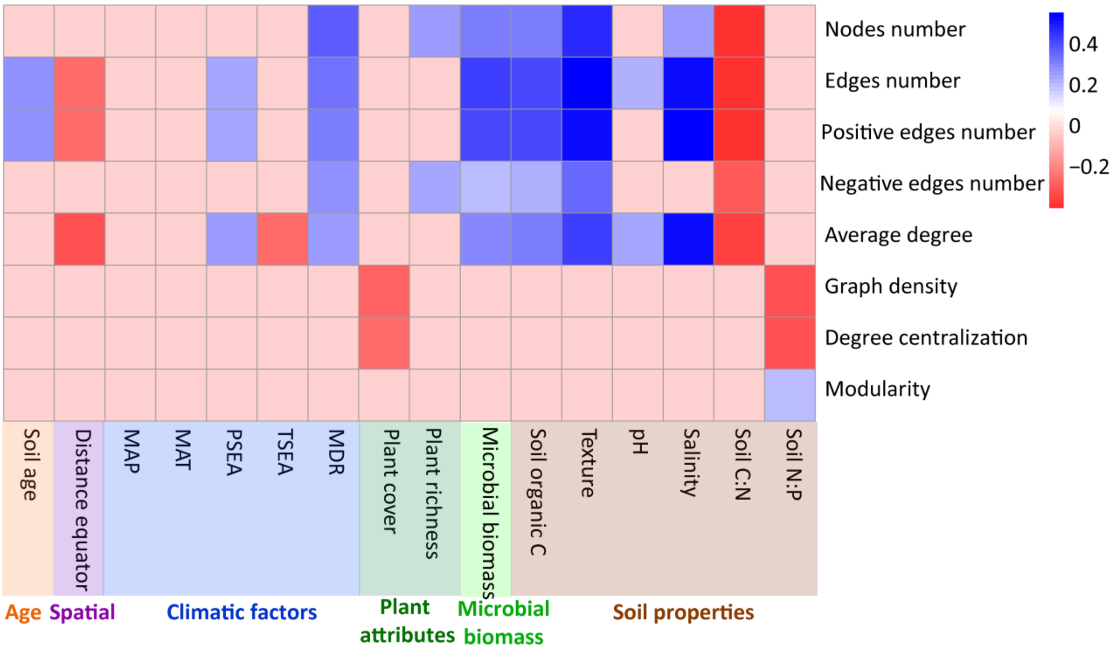

Figure S13 | Relationships between environmental (microbial biomass, spatial, climatic, plant and edaphic) factors and microbial network traits across chronosequences. MAP, mean annual precipitation; MAT, mean annual temperature; PSEA, precipitation seasonality; TSEA, temperature seasonality; MDR, mean diurnal range. Source data are provided as a Source Data file.

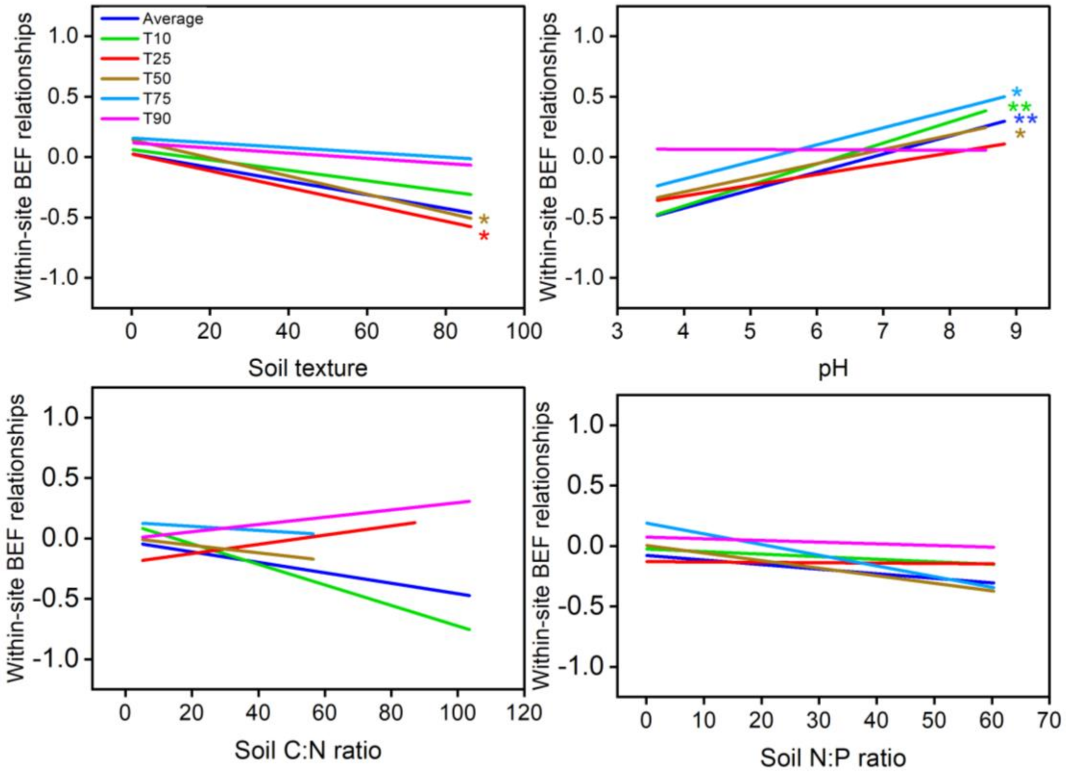

Figure S14 | Potential edaphic factors regulating the local (within-site) multidiversity and ecosystem function (BEF) relationships. Average represents multifunctionality qualified by averaging all 13 individual functions after 0-1 standardization (Methods); T10, T25, T50, T75 and T90, represents multifunctionality quantified at threshold of 10%, 25%, 75% and 90%, respectively. *, $P < 0.05$; ** $P < 0.01$. Source data are provided as a Source Data file.

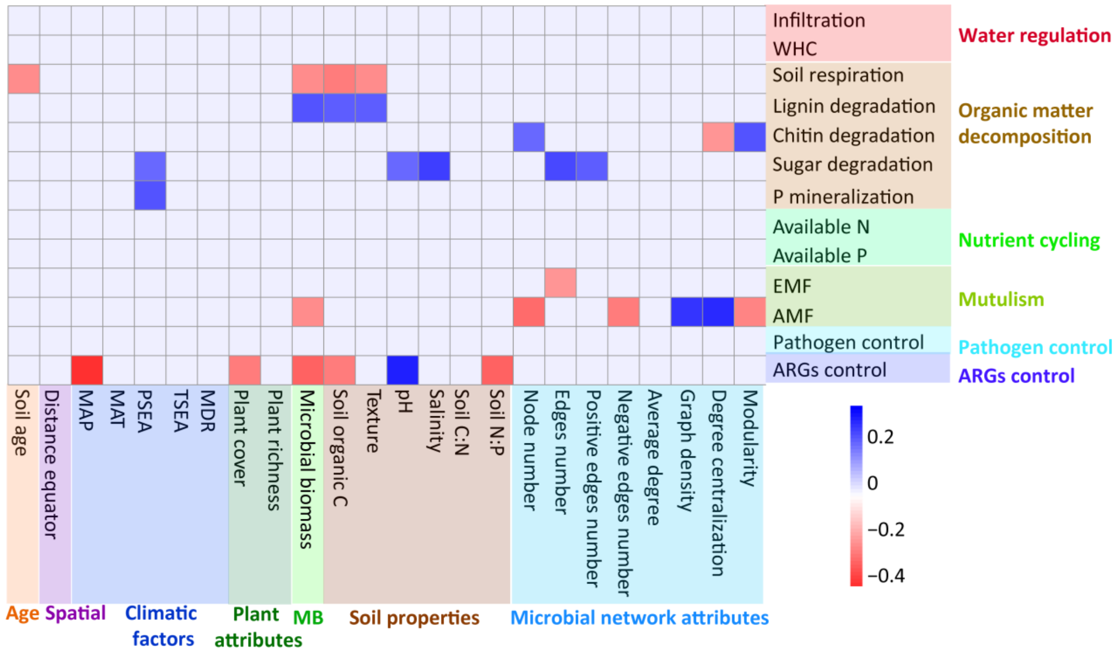

Figure S15 | Potential environmental factors (spatial, climatic, plant, edaphic, and microbial factors and microbial network traits) regulating the local (within-site) multidiversity and individual ecosystem function relationships. MAP, mean annual precipitation; MAT, mean annual temperature; PSEA, precipitation seasonality; TSEA, temperature seasonality; MDR, mean diurnal range. WHC, water holding capacity; EMF, Ectomycorrhizal fungi; AMF, Arbuscular mycorrhizal fungi; ARGs, antibiotic resistance genes; MB, microbial biomass. Source data are provided as a Source Data file.

Table S1 | Detailed information for the 16 chronosequences from 87 plots worldwide. Data was retrieved from ref. 1.

Plot ID	Lable	Name	Age	Biome	Chronosequence origin	MAP	MAT
1	AZ1	SAGA	900	Arid forests	Volcanic	353	11.5
2	AZ2	SAGA	55000	Arid forests	Volcanic	383	11.9
3	AZ3	SAGA	750000	Arid forests	Volcanic	457	8.6
4	AZ4	SAGA	3000000	Arid forests	Volcanic	490	9.5
5	CO1	Coal creek	5000	Cold grasslands	Sedimentary	488	9.7
6	CO2	Coal creek	140000	Cold grasslands	Sedimentary	489	9.7
7	CO3	Coal creek	240000	Cold grasslands	Sedimentary	489	9.7
8	CO4	Coal creek	640000	Cold grasslands	Sedimentary	479	9.3
9	CO5	Coal creek	1000000	Cold grasslands	Sedimentary	473	8.5
10	CO6	Coal creek	2000000	Cold grasslands	Sedimentary	473	8.5
11	CH1	Conguillio	60	Temperate forests	Volcanic	1905	9.5
12	CH5	Conguillio	60000	Temperate forests	Volcanic	1932	9.1
13	CH3	Conguillio	776	Temperate forests	Volcanic	1913	7.9
14	CH4	Conguillio	3470	Temperate forests	Volcanic	1879	7.6
15	CH2	Conguillio	266	Temperate forests	Volcanic	1908	9.3
16	CH6	Conguillio	5000000	Temperate forests	Volcanic	1905	8.8
17	ICE1	Mt Hekla	172	Polar moss heaths	Volcanic	1088	3.7
18	ICE2	Mt Hekla	463	Polar moss heaths	Volcanic	1230	3.6
19	ICE3	Mt Hekla	628	Polar moss heaths	Volcanic	1139	3.3
20	ICE4	Mt Hekla	717	Polar moss heaths	Volcanic	1214	3.6
21	ICE5	Mt Hekla	859	Polar moss heaths	Volcanic	1129	3.4
22	CI1	La Palma	525	Temperate forests	Volcanic	451	14.4
23	CI2	La Palma	6000	Temperate forests	Volcanic	489	12.6
24	CI3	La Palma	40000	Temperate forests	Volcanic	402	16.0

25	CI4	La Palma	600000	Temperate forests	Volcanic	499	11.1
26	CI5	La Palma	1100000	Temperate forests	Volcanic	434	14.2
27	CI6	La Palma	1700000	Temperate forests	Volcanic	433	14.2
28	BOV1	Chiar Kkollu	25	Arid shrublands	Volcanic	81	7.0
29	BOV2	Chiar Kkollu	11400	Arid shrublands	Volcanic	81	6.8
30	BOV3	Chiar Kkollu	14100	Arid shrublands	Volcanic	81	6.8
31	BOV4	Chiar Kkollu	20000	Arid shrublands	Volcanic	81	6.8
32	BOS1	Cojiri	25	Arid shrublands	Sedimentary	131	7.1
33	BOS2	Cojiri	11400	Arid shrublands	Sedimentary	131	7.1
34	BOS3	Cojiri	14100	Arid shrublands	Sedimentary	131	7.1
35	BOS4	Cojiri	20000	Arid shrublands	Sedimentary	126	7.0
36	ALPS1	Alps	10	Alpine ecosystems	Glacier	933	-2.8
37	ALPS2	Alps	45	Alpine ecosystems	Glacier	859	-2.2
38	ALPS3	Alps	125	Alpine ecosystems	Glacier	809	-1.5
39	ALPS4	Alps	10000	Alpine ecosystems	Glacier	826	-1.7
40	ALPS5	Alps	120000	Alpine ecosystems	Glacier	1316	7.9
41	JOR1	Jornada Desert	1100	Arid forblands	Sedimentary	303	15.1
42	JOR2	Jornada Desert	2200	Arid forblands	Sedimentary	275	15.4
43	JOR3	Jornada Desert	8000	Arid forblands	Sedimentary	275	15.4
44	JOR4	Jornada Desert	25000	Arid forblands	Sedimentary	273	15.5
45	WA1	Jurien Bay	100	Temperate shrublands	Sand dunes	565	19.6
46	WA2	Jurien Bay	1000	Temperate shrublands	Sand dunes	555	19.3
47	WA3	Jurien Bay	6500	Temperate shrublands	Sand dunes	557	19.5
48	WA4	Jurien Bay	120000	Temperate shrublands	Sand dunes	556	19.6
49	WA5	Jurien Bay	480000	Temperate shrublands	Sand dunes	555	19.7
50	WA6	Jurien Bay	2000000	Temperate shrublands	Sand dunes	560	19.6

51	QL1	Cooloola	3600	Temperate forests	Sand dunes	1381	20.9
52	QL2	Cooloola	6700	Temperate forests	Sand dunes	1451	20.2
53	QL3	Cooloola	134000	Temperate forests	Sand dunes	1446	20.4
54	QL4	Cooloola	176000	Temperate forests	Sand dunes	1459	20.1
55	QL5	Cooloola	324000	Temperate forests	Sand dunes	1367	21.0
56	QL6	Cooloola	716000	Temperate forests	Sand dunes	1401	20.4
57	MEX1	Chichinautzin	1000	Temperate forests	Volcanic	1056	11.0
58	MEX2	Chichinautzin	1835	Temperate forests	Volcanic	1230	11.7
59	MEX3	Chichinautzin	3800	Temperate forests	Volcanic	1240	12.1
60	MEX4	Chichinautzin	6200	Temperate forests	Volcanic	1209	12.0
61	MEX5	Chichinautzin	8000	Temperate forests	Volcanic	1197	12.1
62	MEX6	Chichinautzin	10000	Temperate forests	Volcanic	1212	11.7
63	MEX7	Chichinautzin	30500	Temperate forests	Volcanic	1203	11.5
64	MEX8	Chichinautzin	100000	Temperate forests	Volcanic	988	10.7
65	MI1	Lake Michigan	73	Cold forests	Sand dunes	778	6.1
66	MI2	Lake Michigan	113	Cold forests	Sand dunes	778	6.1
67	MI3	Lake Michigan	163	Cold forests	Sand dunes	778	6.1
68	MI4	Lake Michigan	243	Cold forests	Sand dunes	778	6.1
69	MI5	Lake Michigan	485	Cold forests	Sand dunes	778	6.1
70	MI6	Lake Michigan	863	Cold forests	Sand dunes	778	6.1
71	MI7	Lake Michigan	1400	Cold forests	Sand dunes	780	6.1
72	MI8	Lake Michigan	2500	Cold forests	Sand dunes	778	6.1
73	MI9	Lake Michigan	3200	Cold forests	Sand dunes	776	6.1
74	MI10	Lake Michigan	4000	Cold forests	Sand dunes	781	6.1
75	HA1	Hawaii	300	Tropical forests	Volcanic	2161	16.4
76	HA2	Hawaii	20000	Tropical forests	Volcanic	2084	15.6

77	HA3	Hawaii	150000	Tropical forests	Volcanic	2085	16.5
78	HA4	Hawaii	4100000	Tropical forests	Volcanic	1248	15.2
79	CAL1	Merced	100	Temperate grasslands	Sedimentary	393	16.3
80	CAL2	Merced	3000	Temperate grasslands	Sedimentary	371	16.0
81	CAL3	Merced	30000	Temperate grasslands	Sedimentary	272	16.1
82	CAL4	Merced	600000	Temperate grasslands	Sedimentary	396	16.3
83	CAL5	Merced	3000000	Temperate grasslands	Sedimentary	462	16.9
84	TA4	Taiwan	399000	Temperate croplands	Sedimentary	2220	21.7
85	TA3	Taiwan	322000	Temperate croplands	Sedimentary	2248	21.7
86	TA2	Taiwan	105000	Temperate croplands	Sedimentary	2347	21.6
87	TA1	Taiwan	28000	Temperate croplands	Sedimentary	2324	21.3

Table S2 | Skewness, kurtosis, and normality tests for local soil biodiversity and ecosystem functions (BEF) relationships.

**Local soil BEF relationship**	**N**	**Skewness**	**$SE_{skewness}$**	**$Z_{skewness}$**	**Kurtosis**	**$SE_{Kurtosis}$**	**$Z_{kurtosis}$**	**Shapiro-Wilk Test** Statistics	*P*-value
Multidiversity vs. Average	80	0.39	0.27	1.45	-1.13	0.53	-2.13	1.10	0.18
Multidiversity vs. T10	72	0.22	0.28	0.78	-1.08	0.56	-1.93	1.17	0.13
Multidiversity vs. T25	68	0.36	0.29	1.22	-0.92	0.57	-1.61	0.81	0.53
Multidiversity vs. T50	67	0.30	0.29	1.01	-1.17	0.58	-2.02	0.88	0.42
Multidiversity vs. T75	54	-0.19	0.33	-0.59	-0.95	0.64	-1.49	0.92	0.36
Multidiversity vs. T90	57	-0.03	0.32	-0.09	-1.27	0.62	-2.04	0.79	0.57
Invertebrate diversity vs. Average	83	-0.01	0.26	-0.04	-1.19	0.52	-2.27	0.79	0.56
Invertebrate diversity vs. T10	75	-0.16	0.28	-0.56	-0.99	0.55	-1.81	0.84	0.48
Invertebrate diversity vs. T25	73	-0.10	0.28	-0.37	-1.03	0.56	-1.86	0.76	0.61
Invertebrate diversity vs. T50	71	0.04	0.29	0.13	-1.22	0.56	-2.16	0.96	0.31
Invertebrate diversity vs. T75	61	-0.24	0.31	-0.78	-0.97	0.60	-1.60	1.06	0.21
Invertebrate diversity vs. T90	63	-0.91	0.30	-3.01	-1.19	0.60	-2.00	0.82	0.52
Protist diversity vs. Average	80	-0.12	0.27	-0.44	-1.39	0.53	-2.61	1.48	0.03
Protist diversity vs. T10	72	-0.03	0.28	-0.10	-1.09	0.56	-1.94	0.80	0.55
Protist diversity vs. T25	70	-0.05	0.29	-0.17	-1.09	0.57	-1.93	0.95	0.33
Protist diversity vs. T50	71	-0.06	0.29	-0.22	-1.45	0.56	-2.57	1.10	0.18
Protist diversity vs. T75	57	-0.23	0.32	-0.72	-0.71	0.62	-1.14	1.26	0.08
Protist diversity vs. T90	65	-0.10	0.30	-0.32	-1.14	0.59	-1.95	0.95	0.32
Fungal diversity vs. Average	85	0.01	0.26	0.02	-0.74	0.52	-1.43	0.67	0.76
Fungal diversity vs. T10	78	0.04	0.27	0.14	-1.12	0.54	-2.07	0.82	0.51
Fungal diversity vs. T25	75	0.12	0.28	0.43	-0.96	0.55	-1.75	0.67	0.74
Fungal diversity vs. T50	75	-0.09	0.28	-0.34	-1.18	0.55	-2.16	1.03	0.24
Fungal diversity vs. T75	63	-0.16	0.30	-0.54	-0.78	0.60	-1.31	0.82	0.52

Fungal diversity vs. T90	72	0.01	0.28	0.05	-1.07	0.56	-1.92	1.12	0.16
Bacterial diversity vs. Average	86	0.14	0.26	0.55	-0.96	0.51	-1.88	0.88	0.42
Bacterial diversity vs. T10	79	0.00	0.27	0.01	-1.05	0.54	-1.95	0.97	0.30
Bacterial diversity vs. T25	77	-0.04	0.27	-0.14	-0.98	0.54	-1.81	0.69	0.74
Bacterial diversity vs. T50	78	0.09	0.27	0.33	-1.19	0.54	-2.21	1.14	0.15
Bacterial diversity vs. T75	66	0.20	0.30	0.68	-0.96	0.58	-1.65	0.78	0.58
Bacterial diversity vs. T90	72	0.17	0.28	0.61	-0.97	0.56	-1.74	0.84	0.49

Notes: Average represents Multifunctionality qualified by averaging all individual 13 individual functions after 0-1 standardization (Methods); T10, T25, T50, T75 and T90, represents multifunctionality quantified at threshold of 10%, 25%, 75% and 90%, respectively. A two-sided test was used to assess whether the data deviated significantly from a normal distribution in either direction. Statistical significance was determined at a threshold of $P < 0.05$.

Supplementary References

1. Delgado-Baquerizo M., *et al.* Changes in belowground biodiversity during ecosystem development. *Proc. Natl. Acad. Sci.* **116**, 6891-6896 (2019).
